# Editorial: Cutting-edge liver surgery-based modalities for diagnosis and treatment of liver tumors

**DOI:** 10.3389/fonc.2023.1256974

**Published:** 2023-08-04

**Authors:** Haoran Hu, Yiyun Gao, Xinyu Zhan, Ye Zhang, Haoming Zhou

**Affiliations:** Hepatobiliary Center, The First Affiliated Hospital of Nanjing Medical University, Key Laboratory of Liver Transplantation, Chinese Academy of Medical Sciences, National Health Commission of the PRC (NHC) Key Laboratory of Living Donor Liver Transplantation (Nanjing Medical University), Nanjing, Jiangsu, China

**Keywords:** liver surgery, laparoscopic liver resection, prognosis prediction, complication treatment, perioperative management

Liver cancer is a common type of malignant tumor in digestive system with high morbidity and mortality worldwide. While surgical resection remains the principal treatment choice, accurate preoperative evaluation, individualized surgical planning, standardized surgical procedures, and appropriate perioperative management are crucial for the prognosis of liver cancer after surgery. This Research Topic embodies 9 multidisciplinary manuscripts focused on multifaceted aspects related to “*Cutting-edge liver surgery-based modalities for diagnosis and treatment of liver tumors*.”

With the development of laparoscopic technique and equipment, laparoscopic liver resection (LLR) has been widely applied at present. To further determine the long-term outcome of LLR for hepatocellular carcinoma (HCCs), Tian et al.‘s group conducted a retrospective study with 1773 HCC patients included and found that LLR for HCCs showed better short-term outcomes and comparable long-term outcomes with laparoscopic liver resection (OLR). Moreover, Xi et al. developed a novel difficulty scoring system to predict the surgical difficulty of LLR, which can help the surgeons to improve the surgical plan and safety. It will become a future trend that using laparoscopic technique in the treatment of different liver tumors.

Both proper hepatic inflow occlusion and hepatic venous system hemorrhage control are essential for the safety of liver resection. Qu et al.‘s group retrospectively analyzed and shared their experience of dealing with Intraoperative hepatic venous system hemorrhage and carbon dioxide gas embolism during LLR. Shi et al. found that while regional and intermittent hepatic inflow occlusion are equally safe and effective, the former showed more advantageous in operation continuity, intraoperative bleeding, and postoperative liver function recovery in LLR. Zhao et al.‘s group introduced the technique of counterclockwise modular laparoscopic anatomic mesohepatectomy using combined Glissonean pedicle and hepatic vein-guided approaches. Besides low central venous pressure technique, Wang X. et al. reported that application of dexmedetomidine during anesthesia improved liver function post hepatectomy. As technology advances, injuries during liver surgery will be better controlled and more patients could be benefited.

In addition to these technical improvements, prognostic factors of liver cancers were also analyzed. Wang, Q. et al. conducted a systematic review and reported that sarcopenia prevalent in patients undergoing PVE/ALPPS might be a risk factor for impaired liver growth. Ge et al. found that both the neutrophil-to-lymphocyte ratio and platelet-to-lymphocyte ratio could be used to effectively predict long-term survival in patients with perihilar cholangiocarcinoma who underwent curative resection. Sun et al.‘s group identified adjuvant TACE timing after radical resection as an independent prognostic factor for patients with HCCs. Effective prognostic prediction is also helpful for the formulation of clinical intervention strategies.

Altogether, the original articles and reviews collected in this Research Topic provide new insights on important achievements obtained in therapeutic strategies, surgical procedure, perioperative management, and analysis of prognostic factors of liver cancers.

## Author contributions

HH: Writing – review & editing, Investigation. YG: Writing – review & editing. XZ: Writing – review & editing. YZ: Writing – review & editing. HZ: Writing – original draft, Writing – review & editing, Supervision

